# What Is New in Pulmonary Mucormycosis?

**DOI:** 10.3390/jof9030307

**Published:** 2023-02-28

**Authors:** François Danion, Anne Coste, Coralie Le Hyaric, Clea Melenotte, Frederic Lamoth, Thierry Calandra, Dea Garcia-Hermoso, Vishukumar Aimanianda, Fanny Lanternier, Olivier Lortholary

**Affiliations:** 1 Service de Maladies Infectieuses et Tropicales, CHU de Strasbourg, Université de Strasbourg, 67000 Strasbourg, France; 2 Laboratoire d’ImmunoRhumatologie Moléculaire, Inserm UMR_S 1109, 67000 Strasbourg, France; 3 Service de Maladies Infectieuses et Tropicales, Centre Hospitalier Régional et Universitaire de Brest, 29200 Brest, France; 4 Inserm LaTIM, UMR 1101, Université de Bretagne Occidentale, 29200 Brest, France; 5 Service de Maladies Infectieuses et Tropicales, Hôpital Necker-Enfants Malades, Assistance Publique-Hôpitaux de Paris, 75015 Paris, France; 6 Infectious Diseases Service, Department of Medicine, Lausanne University Hospital and University of Lausanne, 1011 Lausanne, Switzerland; 7 Institute of Microbiology, Lausanne University Hospital and University of Lausanne, 1011 Lausanne, Switzerland; 8 Service of Immunology and Allergy, Department of Medicine and Department of Laboratory Medicine and Pathology, Lausanne University Hospital and University of Lausanne, 1011 Lausanne, Switzerland; 9 CNR Mycoses Invasives, Groupe de Recherche Mycologie Translationnelle, Institut Pasteur, Université Paris Cité, 75015 Paris, France

**Keywords:** pulmonary mucormycosis, Mucorales, review, Epidemiology, treatment

## Abstract

Mucormycosis is a rare but life-threatening fungal infection due to molds of the order Mucorales. The incidence has been increasing over recent decades. Worldwide, pulmonary mucormycosis (PM) presents in the lungs, which are the third main location for the infection after the rhino-orbito-cerebral (ROC) areas and the skin. The main risk factors for PM include hematological malignancies and solid organ transplantation, whereas ROC infections classically are classically favored by diabetes mellitus. The differences between the ROC and pulmonary locations are possibly explained by the activation of different mammalian receptors—GRP78 in nasal epithelial cells and integrin β1 in alveolar epithelial cells—in response to Mucorales. Alveolar macrophages and neutrophils play a key role in the host defense against Mucorales. The diagnosis of PM relies on CT scans, cultures, PCR tests, and histology. The reversed halo sign is an early, but very suggestive, sign of PM in neutropenic patients. Recently, the serum PCR test showed a very encouraging performance for the diagnosis and follow-up of mucormycosis. Liposomal amphotericin B is the drug of choice for first-line therapy, together with correction of underlying disease and surgery when feasible. After a stable or partial response, the step-down treatment includes oral isavuconazole or posaconazole delayed release tablets until a complete response is achieved. Secondary prophylaxis should be discussed when there is any risk of relapse, such as the persistence of neutropenia or the prolonged use of high-dose immunosuppressive therapy. Despite these novelties, the mortality rate from PM remains higher than 50%. Therefore, future research must define the place for combination therapy and adjunctive treatments, while the development of new treatments is necessary.

## 1. Introduction

Mucormycosis is a rare but life-threatening invasive fungal infection caused by filamentous fungi of the order Mucorales. The incidence of mucormycosis has increased from 0.07 to 0.12 per 100,000 population during 2001–2010 in France; currently mucormycosis is the fourth most common cause of invasive fungal disease after fungemia, pneumocystosis and invasive aspergillosis [1,2]. This trend of increasing mucormycosis has also been reported by other European countries such as Spain, Switzerland, and Belgium [3,4,5,6]. An active surveillance program by the French surveillance network of invasive fungal infections (RESSIF) has reported the incidence of mucormycosis remained stable between 2012 and 2018 [2]. In an autopsy series, mucormycosis had a frequency of 1–5 cases per 10,000 autopsies, which was 10–50 folds less frequent than invasive candidiasis or aspergillosis [7]. It is important to mention that the incidence of mucormycosis is not well documented, as this rare fungal infection is not a reportable disease and is difficult to diagnose because the number of autopsies performed is decreasing [8].

The incidence is more pronounced in India, where it is nearly 70 times higher than in the rest of the world. The incidence in India increased from 1990 to 2015 [9,10]. Rhino-orbito-cerebral (ROC) forms are predominant in this country and mostly related to diabetes mellitus (DM). DM has become a public health threat with a prevalence in adults (20–79 years) estimated to affect 537 million people worldwide in 2021, and will rise to 783 million in 2045 [11]. In India, this number is estimated to be 74 million in 2021 and predicted to rise to 125 million in 2045 [11]. Finally, the COVID-19 pandemic was associated with an important increase of COVID-associated mucormycosis (CAM) cases in India between May and July 2021. Mucormycosis became a notifiable disease in India in May 2021, with over 47,000 reported cases during the following three months [12].

The increased incidence over the last decades and the emergence of CAM, which has been widely reported in the media as “the black fungus threat” in India, brought mucormycosis to the attention of the public and health authorities. In the present narrative review on PM, we discuss recent updates on risk factors, physiopathology, diagnosis, and treatment.

## 2. Risk Factors

**Figure 1:**
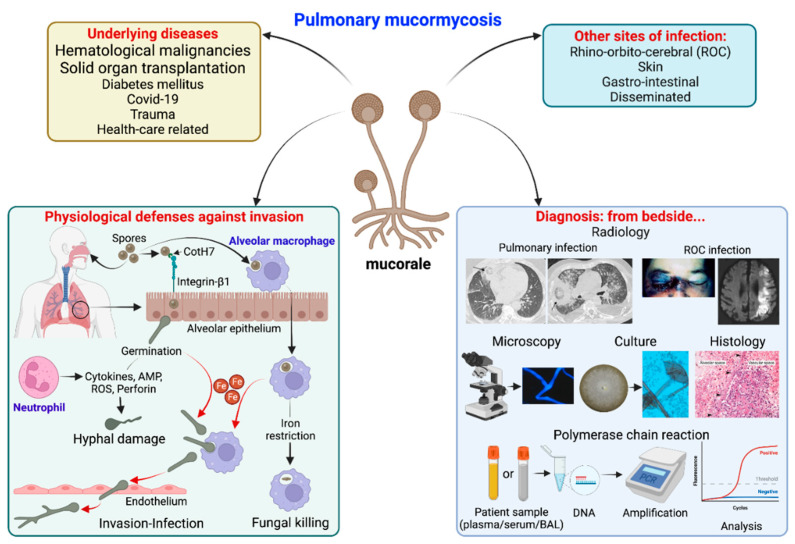
Summary of pulmonary mucormycosis: underlying diseases, sites of infection, physiological defenses against pulmonary invasion and diagnostic methods from the bedside. Physiological defenses in brief: alveolar macrophages can uptake Mucorales spores and restrict iron (Fe), which is essential for spore germination, resulting in spore persistence inside macrophages or their killing by defense mechanisms. On the other hand, integrin-β1 expressed on alveolar epithelial cells can recognize CotH7 on spores, facilitating spore internalization-germination. These germinating spores are recognized by neutrophils, which produce defensive molecules [cytokines, antimicrobial peptides (AMP), reactive oxygen species (ROS) or perforins] that result in hyphal damage. On the other hand, spores germinate in presence of Fe (both inside and outside macrophages) and invade endothelial cells resulting in invasion.

Pulmonary mucormycosis (PM) mainly occurs in immunocompromised patients (Figure 1). Hematological malignancies (HM) and solid organ transplantations are classical risk factors [7]. It is now well known that the sites of the Mucorales infection depend on the underlying disease. Indeed, HM are associated with pulmonary or disseminated mucormycosis, whereas DM is most often associated with the ROC location [7,13,14].

The main risk factor for PM is HM. Three studies conducted in North America, Europe, and France showed that among patients with PM, the proportion of patients with HM was high, from 50 to 80% [13,15,16]. Inversely, among patients with HM, the lung was the main site of involvement (between 34–44%). The association of PM and HM was recently confirmed in a study focusing on 114 PM cases in six tertiary centers in France during the period 2008–2019 [17]. HM were the main underlying conditions (70%), including patients with acute myeloid leukemia (24%), hematopoietic stem cell transplant (HSCT) recipients (21%), and patients with lymphoma (13%).

Solid organ transplantation (SOT) is the second most common risk factor for PM. Most PM cases occur during the first year post-transplant [18]. In the recent collaborative French series on PM, SOT represented 17% of the population [17]. Inversely, among patients with SOT, the lung was the predominant location. PM was documented in half of these patients (53%) in a study conducted between 2005 and 2008 [18,19]. In another series of 116 patients with SOT and mucormycosis, PM was also predominant (24%) [20]. Among the 28 patients with SOT, the organs transplanted were the kidney, the heart, and the lung in 68%, 25%, and 7% cases, respectively.

COVID-19 now appears to be a risk factor for mucormycosis in patients with DM, especially in India. Uncontrolled DM and the use of dexamethasone, recommended in the treatment of COVID-19 requiring oxygen, were major additional risk factors for CAM [12,21]. Interestingly, the cumulative glucocorticoid dose was more important in India than in the rest of the world. ROC locations were largely predominant [22,23]. Primary PM was diagnosed in only nine percent of cases, but was predictive of death with an OR of 3.2 (1.05–10.11) compared to rhino-orbital forms [22]. In high income countries, COVID-19 was not associated with an increase of mucormycosis cases, and the presentation differed from that in India [12,24,25]. In France, 17 cases have been reported; 16 (94%) were hospitalized in the ICU [24]. Of these, 47% had diabetes mellitus and 35% had hematological malignancies. PM was predominant (56%), while 18% had disseminated infection. Thirteen patients (76%) received corticosteroids. Recommendations on the management of CAM have been developed by Indian experts [26].

DM and ketoacidosis are important risk factors, notably for ROC disease. The prevalence of DM-related mucormycosis has geographical variations. DM was more often reported in Asian (46%) and African countries (75%) compared to Western countries (36%) [14]. In the recent French series on PM, only 18% of the patients had DM, which was the sole risk factor for five patients (4%) [17].

Cases of PM have recently been reported in 21 patients who had pulmonary tuberculosis, representing five percent of the 388 cases of this cohort of mucormycosis [27].

Trauma is another risk factor, mainly for cutaneous mucormycosis [28]. A few pulmonary cases have been reported after multiple traumatic injuries [29,30]. Hurricanes that are followed by major flooding have been associated with the development of extensive mold growth in flood-damaged homes [31,32]. Eight patients were identified with transient respiratory colonization with *Syncephalastrum* after hurricanes Katrina and Rita in 2005 in the USA [33].

Healthcare-associated mucormycosis is rarely associated with primary lung involvement, but mainly with skin locations. In a review of the literature, PM accounts for six percent of health-care associated mucormycosis cases with an identified or suspected source of infection [34]. Lung transplantation was a risk factor, and mucormycosis involved bronchial anastomosis.

Breakthrough mucormycosis has been frequently reported with voriconazole, which has no anti-Mucorales effects. This treatment appeared as an independent risk-factor for mucormycosis in case-controlled studies of HM patients [19,35,36]. Whether voriconazole decreases the risk of aspergillosis resulting in the emergence of rare fungi or if it is a true risk factor is still a matter of debate.

Few data are available regarding pediatric populations [37,38,39]. In a systematic review of 157 pediatric cases of mucormycosis from the literature, the main underlying conditions were neutropenia (18%) and prematurity (17%). PM represented 16% of the cases [38]. In the USA, a retrospective study included 156 children [37]. The incidence of mucormycosis was stable over the period 2003–2010, reaching 3.5 infections per 100,000 discharges in 2010. The median age was 10 years, and the main underlying disease was malignancy in 58%. In-hospital mortality was 25%. A recent series from two registries of European and non-European countries included 63 cases of mucormycosis in children. PM was diagnosed in 19% and disseminated forms in 38%. In PM, HM (58%) and HSCT (25%) were predominant, and the mortality rate was 30% [39].

**What should be remembered?** HM and SOT are the main risk factors for PM, whereas DM increases the susceptibility to ROC mucormycosis. A peak of COVID-19 associated mucormycosis occurred in May 2021 in India, involving mainly ROC forms.

## 3. Pathophysiology

Mucorales spores are ubiquitous in nature, found in the soil and in decaying organic matter. Spores are airborne, leading to ROC, mostly in diabetic patients or to pulmonary form upon their inhalation in patients with HM or SOT. For a long time, the question was why there was such a difference in tissue targets. This difference has recently been explained, at least in part, by the activation of various mammalian receptors depending on the host cell type as nicely evidenced through the contribution of a UCLA group [40].

First, the glucose-regulated protein 78 (GRP78), an endoplasmic reticulum chaperone protein of the HSP70 family, induced notably by an elevated level of glucose, has been identified as the host receptor for *Rhizopus arrhizus* in endothelial cells in diabetic mice [41]. Fungal coat protein homologs 3 (CotH3) was identified as the ligand for GRP78 [42].

Afterwards, it was shown that the fungal CotH3 protein interacts with GRP78 on nasal epithelial cells as well [40] (Figure 1). Elevated levels of glucose, iron, and ketones, which are hallmarks of ketoacidosis, induce the expression of GRP78 and CotH3, enhancing the invasion and damage of these cells. Antibodies against CotH3 or GRP78 decreased the invasion and damage [43]. In a recent study, serum GRP78 levels were significantly higher in CAM than in COVID-19 controls. Further studies are required to better assess the role of GRP78 in the pathogenesis of CAM [44].

On the other hand, fungal CotH7 recognizes integrin β1 as the receptor on alveolar epithelial cells. Integrins are highly expressed in human lung tissues. This binding triggers the activation of epidermal growth factor receptor (EGFR) signaling, leading to host cell invasion and pulmonary infection [40]. Interestingly, anti-integrin antibodies protect neutropenic mice from the infection.

Alveolar macrophages have a predominant role in the host defense against Mucorales, through the control of spore proliferation, as demonstrated by Andrianaki et al. [45]. First, the specific ablation of alveolar macrophages increased susceptibility to the infection. Moreover, the spores of Mucorales are phagocytosed by macrophages but not by neutrophils. Interestingly, these spores remained viable inside alveolar macrophages for at least 10 days post-infection. The mechanism of defense used by alveolar macrophages was the inhibition of intracellular swelling of conidia of *Rhizopus* by iron starvation [45]. This nutritional immunity is important, as iron is essential for the Mucorales life cycle. There is a specific susceptibility to mucormycosis in patients with ketoacidosis and in patients in dialysis where deferoxamine is used. Indeed, there is increased availability of free iron in ketoacidosis, while the fungus uses deferoxamine as a siderophore [45,46,47]. Hyperglycemia, acidosis, and steroids are, indeed, classical risk factors for mucormycosis, which impair the phagocytic function of alveolar macrophages, thereby decreasing host defenses against Mucorales [48,49,50]. Alveolar macrophages from mice pretreated with corticosteroids had higher fungal growth due to phagocytosis inhibition and reduced oxidative burst [48]. In the *Drosophila* model, corticosteroids, increased iron supply, and iron availability increased the pathogenicity of mucormycosis, as in humans [51].

The importance of neutrophils is first shown by the fact that neutropenia is a major risk factor for mucormycosis. Neutrophils recognize Mucorales mainly by TLR2, leading to the production of pro-inflammatory cytokines [52,53]. These cytokines damage both spores and hyphae of the fungus by inducing production of reactive oxygen metabolites, cationic peptides and perforin [54]. By comparison, neutrophils damage *R. arrhizus* than *A. fumigatus* less efficiently. Differences have also been noted between the species of Mucorales. Interestingly, the production of oxidative burst is lower in response to the *Rhizopus* species compared to that recorded with *Lichtheimia corymbifera* [55].

The development of mucormycosis in chronic granulomatous diseases (CGD) reinforces the understanding of the role of neutrophils in the defense against Mucorales. The mutation in the genes encoding the nicotinamide adenine dinucleotide phosphate (NADPH) oxidase, responsible for CGD, impairs the production of reactive oxygen species by neutrophils. Few cases or small case-series have reported mucormycosis [56,57,58,59,60,61,62]. Notably, most of the mucormycosis patients also received steroids and had PM. Few cases of mucormycosis have also been reported among other primary immune deficiencies, including caspase recruitment domain-containing protein 9 (CARD9) deficiency and STAT1 gain-of-function [63,64]. Finally, neutrophil responses and T Helper cell were impaired in CARD9 knockout mice in a model of subcutaneous *Mucor irregularis* infection [65].

**What should be remembered**? Alveolar macrophages and neutrophils are major innate immune cells involved in the defense against Mucorales. The differences between ROC and pulmonary locations are explained at least in part by the activation of different mammalian receptors, GRP78 in nasal epithelial cells and integrin β1 in alveolar epithelial cells, in response to Mucorales invasion.

## 4. Clinical and Radiological Findings

PM is the third most common site of infection (20%), after ROC (34%) and cutaneous (22%) mucormycosis in a review of 600 published articles in the literature [14].

Symptoms of PM are non-specific. Persistent fever despite broad spectrum antibiotics and pulmonary symptoms are suggestive of a pulmonary fungal invasive infection in a patient at risk [66]. These symptoms include cough, dyspnea, chest pain, or hemoptysis. Hemoptysis is described in 13 to 31% of the different series on PM [17,66,67,68,69]. Some patients might be asymptomatic [70]. In the presence of PM, clinicians should search for other clinical involvement such as ROC, skin, or digestive symptoms. Gastro-intestinal symptoms are non-specific and mainly include abdominal pain, gastrointestinal bleeding, and change in bowel habits [28].

PM can have endobronchial involvement. Among 35 patients with HM and PM who underwent fiberoptic bronchoscopy, 34 had visible endobronchial diseases. The main findings were stenosis (24%), erythematous mucosa (18%), obstruction of airway (12%), gelatinous or mucoid secretions (12, and polypoid mass (12%) [66]. Endobronchial and parenchyma involvement can coexist but tracheobronchial mucormycosis without parenchyma involvement are mainly seen in diabetic patients [71].

**Figure 2:**
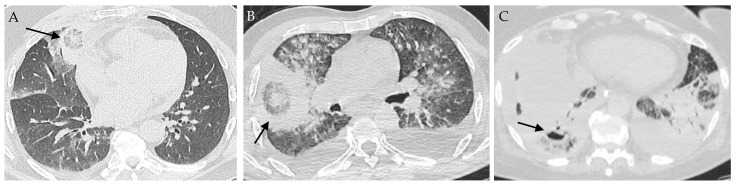
Images of pulmonary mucormycosis. (**A**) Reversed halo sign (indicated with an arrow) in a patient with hematological malignancy; (**B**) Reversed halo sign HS (arrow) in a patient with COVID-19; (**C**) Cavitation (arrow) in a condensation in a patient with COVID-19 and lung tumor.

Chest computerized tomography (CT)-scan is recommended in patients with a suspicion of pulmonary fungal infection. The presence of a reversed halo sign (RHS) is highly suggestive of PM [67,72]. It is a focal ground-glass opacity associated with a ring or crescent-shaped consolidation (Figure 2). The central ground glass opacity corresponds to coagulative necrosis whereas the peripheral condensation is associated with liquefaction, consolidation, and organization. [73]. The frequency of RHS varied from 19% to 95% and depended on the underlying disease and the timing of imaging [67,74]. Legouge et al. reported RHS in 15 out of 16 patients with proven PM and neutropenia [67]. Coste et al. identified RHS in 26% of the 114 patients with PM, but this proportion increased to 40% in patients with HM [17]. Finally, the RHS is an early sign, which disappeared after day 15 in the study from Legouge et al. [67].

Other findings are consolidation, nodules, halo sign, micronodules, mass, cavitation, and pleural effusion, but these are non-specific. In the recent retrospective French study on PM, condensation and ground glass opacities were the predominant lesions (58% and 65%, respectively) [17]. Neutropenic patients presented more frequently than non-neutropenic patients with ground-glass opacities (75 vs. 49%), halo sign (32% vs. 10%), and reversed halo sign (35 vs. 10%). Vessel occlusion is a sensitive sign for the diagnosis of invasive mold disease in patients with HM, but was not specifically studied for mucormycosis [75,76].

One of the difficulties is to find radiologic features suggesting PM rather than pulmonary aspergillosis as they share the same risk factors and clinical presentation but may not require the same treatments. The presence of more than 10 nodules (OR, 19.82 [95% CI: 1.94, 202.29]) and of pleural effusion (OR 5.1 [95% CI: 1.06, 24.23]) were predictors of PM compared to pulmonary aspergillosis in a retrospective study of patients with cancer [68]. Another retrospective study comparing these two pulmonary fungal infections showed more RHS in patients with PM (54% vs. 6%) compared to pulmonary aspergillosis, but less airway-invasive features, including clusters of centrilobular nodules (29% vs. 52%), peri-bronchial consolidations (21% vs. 49%), and bronchial wall thickening (4% vs. 34%) [77].

In patients with suspected or confirmed PM, other locations should be systematically investigated. Sinus involvement is frequently reported in patients with PM; the reported frequencies varied from 13% to 95% of the cases [17,68,78]. This association of sinus and lung infections is more frequent in mucormycosis than in aspergillosis. Patients with PM, especially when they are neutropenic, present with a disseminated form in 16–40% of cases [17,18,78,79]. Main sites of dissemination include liver (48%), spleen (48%), brain (44%), kidneys, and skin [17]. Several patients with premortem diagnosis of PM have occult disseminated disease at autopsy [80]. The research on brain dissemination is of particular importance, as the treatment of brain mucormycosis differs from other locations. Other sites of infection, such as the skin, can contribute to the diagnosis of mucormycosis by offering an easily accessible biopsy site. Finally, in view of the high frequency of occult dissemination and the early angioinvasive nature of infections, CTs of sinuses, abdomen, and pelvis are needed to research other locations of PM and stage the disease [80,81]. Magnetic resonance imaging is recommended if there is a suspicion of brain, sinus or eye disease [82].

18F-Fluorodeoxyglucose positron emission tomography CT (PET/CT) has shown interesting results in small series for the diagnosis, the staging and the treatment’s duration [83,84,85]. Prospective studies are needed to decide the adequate place of this contributive tool.

**What should be remembered?** A reversed halo sign is an early and transient, but very suggestive, sign of PM in neutropenic patients. Other locations of mucormycosis should be evaluated with a body CT-scan and cerebral imaging.

## 5. Diagnostic Tests

The three most frequent human pathogens in mucormycosis are the species of *Rhizopus*, *Mucor* and *Lichtheimia*, followed by *Apophysomyces*, *Cunninghamella*, *Rhizomucor*, and *Saksenaea* [14,82]. In the recent French surveillance on PM, the species of *Rhizomucor*, *Rhizopus*, and *Lichtheimia* were predominantly identified in 32%, 30%, and 25%, respectively [17]. Species of *Cunninghamella* were more common in patients with PM or with disseminated forms, whereas that of *Rhizopus* was more frequent in ROC mucormycosis [14]. *Saksenaea* and *Apophysomyces* were mostly associated with cutaneous mucormycosis, but not PM. There are also geographical variations: *Lichtheimia* species were predominantly documented in Europe, whereas *Apophysomyces* species were not observed in Europe or Africa.

The diagnosis of mucormycosis is made harder to establish by the existence of mixed mold infections. In a series of 690 patients with invasive aspergillosis, 25 of them (4%) had concomitant mucormycosis [86]. Among the 40 patients with mucormycosis in the Modimucor study, 32% of them presented with *Aspergillus* coinfection [87].

Mucorales are identified by culture and non-culture methods from specimens, such as tissue biopsy, broncho-alveolar lavage (BAL) fluid or other respiratory samples, and serum. Every effort should be made to obtain specimens. For PM, bronchial endoscopy with bronchial aspiration and BAL are needed. Sputum and tracheal aspiration can be realized when it is possible. CT-guided percutaneous biopsy should be performed when BAL is non-diagnostic, but this procedure is contra-indicated in patients with platelets below 50,000/mm^3^ [88]. In a series of 16 patients with PM, diagnosis was made by CT-guided trans-thoracic biopsy (50%), surgical pulmonary resection (31%), cutaneous biopsy (13%) and pleural puncture (6%) [67]. Biopsies of other dissemination sites, such as the skin, can contribute to mucormycosis diagnosis when available.

**Figure 3:**
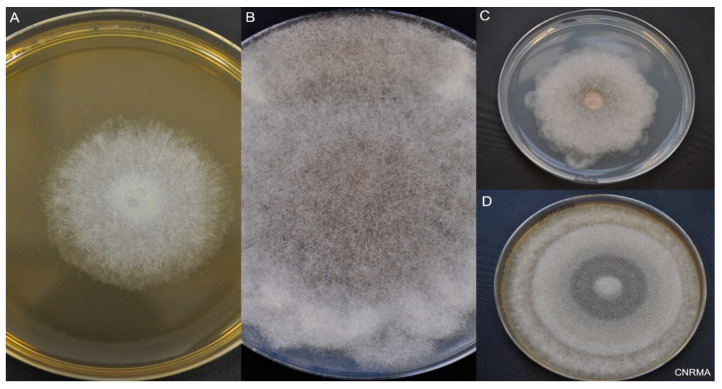
Macroscopic aspect of Mucorales. Incubation at 30 °C on 2% malt extract (MEA) or potato dextrose agar (PDA). (**A**) *Lichtheimia ramosa* on MEA at day 2. (**B**) *Rhizopus microsporus* on PDA at day 6. (**C**) *Lichtheimia corymbifera* on PDA at day 6. (**D**) *Mucor velutinosus* on PDA at day 6.

Mucorales are identified by macroscopic and microscopic examination [89] (Figure 3). The typical morphology of hyphae is large (5–25 µm), ribbon-like, non- or pauci-septated, with an irregular pattern of branching. Recovery of Mucorales from clinical specimens is difficult, with positive culture in only 15 to 25% of cases [90]. However, culture is suitable for the definite diagnosis of mucormycosis. Culture is also of prime importance for identification to the species level, and for antifungal susceptibility testing of the isolate, keeping in mind that there is a lack of MIC-outcome correlation [91,92]. The different species share common morphological characteristics, making it difficult to use phenotypic methods to identify the species. Definite species identification requires molecular methods (principally by the sequencing of the internal transcribed spacer region), or matrix-assisted laser desorption ionization–time of flight mass spectrometry (MALDI-TOF MS) techniques [89].

**Figure 4:**
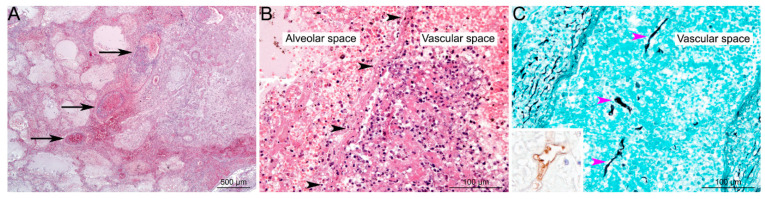
Histological lesions in pulmonary Mucormycosis. (**A**) Focal well-delineated lesion, centred on arteries and veins of medium calibre (black arrows), characterized by vascular alterations associated with peripheral alveolar oedema, acute hemorrhages, ischemic necrosis, and minimal inflammation (pulmonary infarction) (HE staining). (**B**) Higher magnification of vascular lesions: thrombi and destruction of vascular wall (black arrowheads), associated with alveolar hemorrhages, oedema and minimal inflammation (mostly neutrophils) (HE staining). (**C**) Hyaline, pleomorphic hyphae are detected in the lesions; very often invading vascular spaces (pink arrowheads). Hyphae are irregular in size (from 3 to 25 µm in diameter), folded, rarely septate (pauciseptate), branching (at right angles) with a ribbon-like morphology, and could be highlighted using Grocott’s Methenamine Silver staining, or immunohistochemistry (inset; primary antibody: GeneTex GTX39191; WSSA-RA-1). Used with kind permission from G. Jouvion. Reproduced from *New developments in Mucormycosis*, Semin Respir Crit Care Med 2015; 36: 692–705 (Publisher Georg Thieme Verlag KG).

Histology can show hyphae that have the characteristics of Mucorales as described before, using haematoxylin-eosin (HE), periodic acid-Schiff stain (PAS), or Grocott-Gomori’s methenamine-silver (GMS) staining, or both [82] (Figure 4). Immunohistochemistry with commercially available monoclonal antibodies or molecular methods on fresh or fixed tissue help to formally confirm the diagnosis of Mucorales infection and avoid misidentification with *Aspergillus species* [93,94,95]. In case of negative mycological culture, PCR in the tissue should be interpreted only when fungal elements have been detected by histopathology [96]. These non-pigmented hyphae are accompanied classically by hemorrhagic infarction, tissue necrosis, angioinvasion, perineural invasion, and a neutrophilic infiltration in acute lesions [81,90,97]. In chronic lesions, there is pyogranulomatous inflammation. The histological findings are also different depending of the underlying disease [81]. Neutropenic patients more frequently display extensive angioinvasion than non-neutropenic patients, whereas allogenic HSCT patients have more inflammatory necrosis and lower frequency of intra-alveolar hemorrhage than those without HSCT.

A PCR-Based diagnosis in serum has been developed by different teams [98]. A quantitative PCR targeting the Mucorales species *Lichtheimia*, *Rhizomucor* and *Mucor*/*Rhizopus* in serum has shown promising preliminary results [99,100]. It was recently evaluated in the prospective Modimucor study, which included 232 patients with a suspicion of invasive mold infection [87]. The performance of this test was encouraging with a sensitivity of 85% and a specificity of 90%. These results were obtained in patients with mainly pulmonary (40%) or disseminated (33%) locations. Interestingly, the positivity of the PCR was obtained earlier compared to the first positive mycological or histological specimen. Additionally, the negativity of serum Mucorales qPCR after seven days of treatment was associated with a lower mortality. This PCR was also evaluated on BAL fluid from patients with pneumonia and immunocompromised conditions, and showed interesting results [101]. In four out of 24 patients, BAL qPCR was the earliest positive test for PM. Two other PCR assays have been developed and can detect more genera. Commercial semi-quantitative PCR (Mucorgenius^®^, PathoNostics) detects the species from the five most frequent genera, *Rhizopus*, *Mucor*, *Lichtheimia*, *Cunninghamella*, and *Rhizomucor*, but cannot distinguish between them [102]. Springer et al. have a two-step procedure using an in-house assay targeting 18S to detect Mucorales, followed by sequencing to identify the genus [103]. All qPCR assays in serum or BAL need to be standardized [98]. They are not yet included in the EORTC/MSGERC criteria for probable mucormycosis, contrary to the *Aspergillus* PCR [104].

Among biomarkers, β-D-glucan and galactomannan antigen are not present or are present in low amounts in the cell wall of Mucorales. They are not detected in the serum of patients with mucormycosis. Novel diagnostic approaches have recently been developed. The detection of a panfungal disaccharide, which turned out to be trehalose, using mass spectrometry, was detected in the serum of 9/16 patients with mucormycosis [105]. A monoclonal antibody recognizing α-1,6 mannan based on purified mannans of *Mucor*, *Rhizopus*, *Aspergillus*, *Fusarium,* and *Candida* species has been constructed as a lateral flow assay [106]. Another lateral-flow device, based on a monoclonal antibody detecting *Rhizopus arrhizus*, has been set up and is compatible with serum and bronchoalveolar fluid [107]. Another team developed an ELISA to detect *Rhizopus*-specific antigen in serum and BAL fluid. Primary results showed higher level of antigen in patients with mucormycosis compared to controls [108]. *CotH* genes are uniquely present among Mucorales and are potential targets for the diagnosis of mucormycosis. *CotH* genes were detected by PCR in urine samples from mice infected by different species of Mucorales. The sensitivity and specificity were 90% and 100%, respectively. They were also detected in the urine samples of patients with proven mucormycosis [109]. The analysis of volatile metabolites was able to distinguish three species of Mucorales (*R. arrhizus*, *R. delemar* and *R microsporus*) from each other in a murine model. These new biomarkers are promising but need prospective validation in human samples.

**What should be remembered?** Every effort should be made to obtain specimens on which to perform direct examination, cultures, and histopathology. Serum qPCR is a non-invasive sensitive and specific test for the diagnosis of mucormycosis. Novel diagnostic approaches are in development.

## 6. Treatment

The treatment of mucormycosis relies on three key elements, which are antifungal treatment, correction of risk factor(s), and surgery. No prospective comparative studies have been performed in this rare infection and no one has specifically studied the treatment of PM.

Prompt antifungal therapy initiation is critical as delay in initiation is associated with increase in mortality. Delaying the initiation of amphotericin B (AmB) for more than three days increased mortality (72 vs. 33%) [15]. A second study in 70 HM patients showed that initiation of amphotericin B after six days from the diagnosis resulted in a two-fold increase in mortality [110].

Liposomal amphotericin B (L-AmB) is the recommended first-line treatment for mucormycosis at a dose of 5–10 mg/kg/day [82]. Evidence data are based on in vitro susceptibility, murine models, and case-series [91,111]. The Ambizygo trial, a single arm of L-AmB at a dose of 10 mg/kg/day, showed a favorable response at week 12 in 45% of the patients, but there was an increase of creatinine in 40% of the patients [112]. A high dose of L-AmB (10 mg/kg) is recommended in case of central nervous system infection, while 5 mg/kg/day is recommended for PM [82]. Regular monitoring of renal function and electrolytes is necessary, with special caution in diabetic patients.

Isavuconazole is a triazole, active in vitro against a majority of Mucorales. Its in vitro activity appears to be species-specific, and it shows reduced activity against *Mucor* species, notably for *Mucor circinelloides* [92,113,114]. The minimum inhibitory concentrations (MICs) are globally higher than those for posaconazole [114]. The VITAL study, a single-arm open-label trial, published in 2016, showed a mortality of 33% at day 42 among the 21 patients treated by isavuconazole. These results were comparable to 33 historical matched controls treated by liposomal amphotericin B, who experienced a mortality of 39% [115]. Based on this trial, isavuconazole has been approved for the treatment of mucormycosis. To note, preexposure to isavuconazole increased the virulence of Mucorales in Drosophila infection model, and breakthrough mucormycosis has been reported in patients receiving isavuconazole [116,117]. Moreover, patients developing breakthrough mucormycosis while on isavuconazole or posaconazole treatment had a higher mortality rate compared to patients who were not on these azole treatment [118].

Posaconazole is also active in vitro against a majority of Mucorales. Of note, some isolates exhibit high MIC, notably *Mucor circinelloides* [92,113,114]. Posaconazole shows efficacy in salvage therapy [119,120] but very few data are available as first-line therapy [16,121]. On the contrary, breakthrough infections have been reported under posaconazole prophylaxis [118,122,123]. The efficacy of this drug was hampered by the limited bioavailability of oral suspension. A delayed release (DR) tablet and an intravenous formulation have recently been developed that help in the management of mucormycosis; the recommended dose is 300 mg per day, after a loading dose of 300 mg × 2 on day 1. Trough concentrations were higher with DR tablets compared to oral suspension. DR tablets are not affected by food intake [124,125].

The therapeutic range of isavuconazole and posaconazole for mucormycosis is unknown. Therapeutic drug monitoring is recommended for posaconazole therapy due to high intra- and inter-individual variability and non-linear pharmacokinetics. The target concentration is >1 µg/mL [82]. On the other side, hepatotoxicity has been associated with serum levels of >1.8 µg/mL [126]. Therapeutic drug monitoring is not recommended for isavuconazole [82]. Indeed, most of the patients treated with isavuconazole had values >1 µg/mL and no correlation was evidenced between serum levels and efficacy or safety [127,128,129].

Isavuconazole and posaconazole are inhibitors of CYP3A4 resulting in drug-drug interactions. Online websites can be helpful for quantitative prediction of drug-drug interaction. Therefore, dosage of immunosuppressive treatments which are metabolized by cytochrome P450 should be adapted during treatment. Other drug-drug interactions, including membrane transporters inhibition, can occur and therapeutic drug monitoring of immunosuppressive treatments is mandatory.

Among new antifungal drugs, fosmanogepix APX001 targets glycosylphosphatidylinositol-anchored protein maturation by inhibiting the fungal enzyme Gwt1. In vitro antifungal activity of fosmanogepix against most Mucorales is limited (minimum effective concentration (MEC)90, 4–16 µg/mL), but some isolates have low MEC (<1 µg/mL) [130,131]. In vivo, fosmanogepix increased survival of mice infected by *R. arrhizus*, in the same manner as isavuconazole, when tested against infecting strains with both low (0.25 µg/mL) and elevated (4 µg/mL) MECs [132]. Ibrexafungerp and olorofim have no activity against Mucorales [132].

Although it was not recommended in recent guidance, the interest of a combined therapy as first line to improve the survival remains an open question [82,133]. In a retrospective study on 106 patients with HM, the combination treatment, mainly AmB with posaconazole and/or an echinocandin, did not impact survival [134]. Another retrospective study on 101 mucormycosis did not show a benefit of amphotericin-echinocandin combination [135]. The potential use of caspofungin in combined therapy was justified by the inhibition in vitro of *Rhizopus arrhizus* β-1,3-D-glucan synthase and an improvement of survival in mice model but only at low dose [136]. On the other hand, some retrospective clinical studies showed interesting results [137,138,139,140]. AmB and posaconazole, mainly used in second or third line, were associated with a clinical improvement after three months in 59% of the HM with mucormycosis in the SEIFEM and Fungiscope registries [140]. The combined therapy, AmB and caspofungin showed positive results in a limited retrospective series of ROC mucormycosis [138]. The role of combined therapy was also studied in diabetic ketoacidosis or neutropenic mice models of disseminated mucormycosis (Table 1). The combination of AmB and echinocandin improved survival [141,142]. The combination of AmB and azole showed variable results; the combination of L-AmB with posaconazole failed to show a benefit on survival, whereas a combination with isavuconazole increased survival compared to each monotherapy [143,144]. Finally, in a neutropenic murine model infected by *R. arrhizus var delemar*, a species with low MECs to fosmanogepix, mice treated by a combination of fosmanogepix and liposomal amphotericin B had a survival of 70%, greater than mice treated with fosmanogepix (30%), liposomal amphotericin B (35%), or receiving a placebo (5%) [145].

Among adjunctive treatments, deferasirox has failed to demonstrate a benefit, at least in patients with HM [146]. Hyperbaric oxygen has shown some benefit in diabetic patients, but not enough data are available to recommend in routine use [82]. Recombinant human granulocyte-macrophage colony-stimulating factor (GM-CSF) is a cytokine that increases phagocytosis and upregulates nonoxidative pathogen killing and neutrophil oxidative metabolism. In patients with HM and refractory invasive fungal infections, the overall response rate was 80% after GM-CSF treatment [147]. Checkpoint inhibitor is a promising adjunctive therapy. The rationale to use checkpoint inhibitor is the T cell exhaustion, which is somehow defined as a dysfunction of effector T cells after chronic antigen stimulation [148]. Anti-PD-1 and PD-L1 have been successfully tested in a murine model of pulmonary mucormycosis showing better survival, less morbidity, and lower fungal burden in lung tissue. Inhibition of PD-L1 was associated with more favorable immune responses than PD-1 [149]. Some efficacy of anti-PD-1 therapy combined with IFN-γ in the treatment of mucormycosis have been reported in several case reports, but more clinical data are needed [150,151,152,153]. Lastly, a promising adjunctive immunotherapeutic option has been developed. Antibodies raised against peptides of CotH3 protected ketoacidotic and neutropenic mice from mucormycosis caused by the most frequent species [43]. Moreover, they were synergistic with posaconazole and liposomal AmB. Anti-integrin β1 antibodies also inhibited *R. delemar* invasion of alveolar epithelial cells and protected mice from pulmonary mucormycosis [40].

Recent guidance from the European Confederation of Medical Mycology and the Mycoses Study Group Education and Research Consortium recommend a first-line therapy with liposomal amphotericin B [82]. When the disease is stable or has partially responded, it is possible (moderately recommended) to switch to oral therapy including isavuconazole or posaconazole delayed release tablet or to continue with first line treatment [82]. Compared to previous recommendations by the same group in 2013 [154] and ECIL [155], major changes concern the use of isavuconazole or posaconazole. Their intravenous formulations are strongly recommended as induction therapy in case of preexisting renal insufficiency and moderately recommended as first line therapy or in case of salvage therapy. When switching to oral therapy, isavuconazole or posaconazole delayed release tablets are strongly recommended, whereas posaconazole oral suspension is only marginally supported due to lower trough levels. Regarding combination therapy, the guidance says that “there are no definitive data to guide the use of antifungal combination therapy. Combination therapy (polyenes and azoles or polyenes plus echinocandins) can be rationally given due to lack of enhanced toxicity with possible but unproven benefit; however, data are too limited to support this beyond a marginal recommendation”. The most clinically relevant duration of the antifungal treatment is not defined but continuing antifungal therapy should be considered until the disappearance of fungal lesions and the reconstitution of host immune system. Finally, there is no specific therapeutic recommendation for PM compared to the other forms of mucormycosis. Only in case of cerebral involvement, the dose of L-AmB should be increased from 5 mg/kg/d to 10 mg/kg/day. Muthu et al. proposed specific recommendations for the management of PM associated with COVID-19 [26].

**Figure 5:**
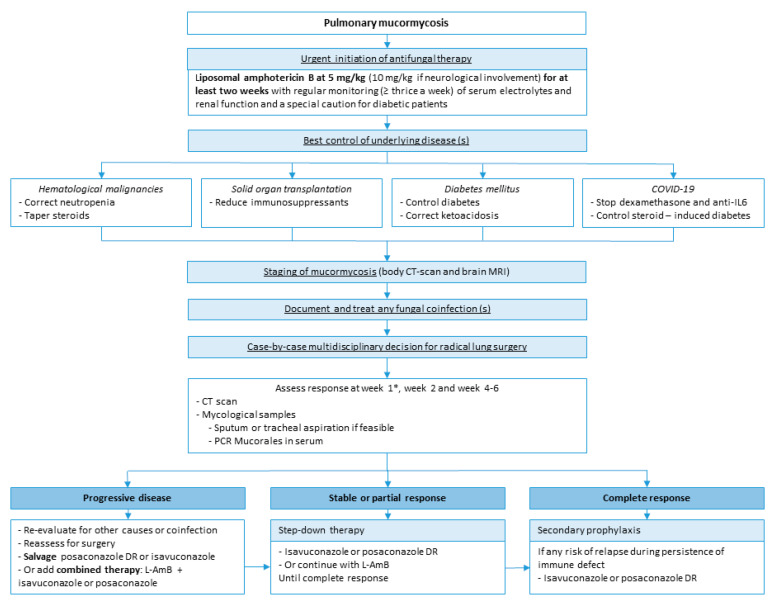
Legend Summary of PM management. * Reevaluation at week 1 should be done with caution. A paradox-ical worsening is possible after neutrophil recovery and should not be considered as a progression of the disease.

**Summary of PM management from our group (Figure 5).**

The initial treatment is based on L-AmB. The correction of risk factors, if feasible, is also a critical point, including control of DM, correction of neutropenia and ketoacidosis, and decrease of immunosuppressants. In COVID-associated mucormycosis, dexamethasone and anti-IL6, given for the viral disease, should be tapered or stopped if possible. Therapeutic response should be assessed by a lung CT scan after one, two and four weeks of treatment and by mycological samples on respiratory samples if feasible and serum PCR. Reevaluation at week 1 should be done with caution; a paradoxical worsening is possible after neutrophil recovery and should not be considered progression of the disease. The duration of the first-line therapy is at least two weeks but may require four weeks or more. After radiological stabilization or partial response, in association with negative mycological samples, step-down therapy with oral isavuconazole or posaconazole can be started. The duration of the treatment is highly variable but needs to be continued until complete clinical and radiological responses. Finally, secondary prophylaxis (i.e., maintenance or suppressive therapy) with oral azole should be discussed when there is any risk of relapse during persistence of immune defect including neutropenia, chemotherapy, or prolonged use of high-dose immunosuppressive therapy. Further studies are warranted to identify risk factors of relapse to better target patients who require prolonged maintenance therapy.

Recent recommendations strongly support an early surgical treatment for mucormycosis in addition to systemic antifungal treatment [82]. Three large series on mucormycosis, not limited to PM, showed a benefit of surgery on improved survival [13,16,79]. On 929 reported cases of mucormycosis, survival was higher in patients treated with antifungal and surgery (70%) than in patients treated with antifungals alone (61%) or surgery alone (57%). In five pulmonary series, mortality was also lower in patients treated with surgery in association with antifungals [66,67,156,157,158]. Despite these results, surgery is not always performed in PM. A recent Korean study has investigated this question. Among 20 cases of PM, nine out of 11 patients who underwent surgery survived whereas one out nine patients without surgery survived. The reasons for not performing surgery were mainly the severity of the underlying disease (refractory underlying disease (*n* = 3), altered mental state (*n* = 1), rapid death after diagnosis (*n* = 1)) and refusal of the patients because of concern for operative risk (*n* = 4) [158]. Moreover, pulmonary surgery might be contraindicated in HM patients with major thrombocytopenia or neutropenia. Finally, patients at high risk of death are less likely to be operated on, limiting the definite conclusions on the right place and the best timing for surgery in PM.

**What should be remembered?** Liposomal amphotericin B is the first line of treatment for mucormycosis in association with the correction of underlying disease and surgery when feasible. Step-down treatment includes oral isavuconazole and posaconazole delayed release tablets.

## 7. Outcome and Prognostic Factors

Mortality depends on the site of involvement in mucormycosis. PM and disseminated forms are associated with lower survival than other forms. In the Retrozygo study, mortality in PM and disseminated forms were 48% and 79% compared to 25 and 22% in rhinocerebral and cutaneous forms, respectively [13]. Mortality in PM varies between 37% to 80% and depends on the underlying disease. Indeed, mortality is more important in patients with HM compared to patients with diabetes mellitus, corresponding to a mortality of 75% and 43% in the study from Lee et al., respectively [13,16,17,66,67,68,78,79,156,159].

APACHE II score, severe lymphocytopenia and high LDH levels have been identified as prognostic factors in patients with HM and PM [78]. Another study of PM in HM patients identified active malignancy and monocytopenia at the time of diagnosis, whereas neutrophil recovery was a protective factor [110]. Finally, the delay of antifungal initiation is a major prognostic factor as shown previously in the therapeutic section.

## 8. Conclusions

Progress has been made in the last decade to improve knowledge of the pathophysiology, the diagnosis, notably via the development of non-invasive PCR in serum, and the treatment of PM. Despite that, mortality remains high; future research must focus on the role of combined therapy, surgery and adjunctive therapy, and the development of new antifungal drugs.

**Table 1:** Combination therapy in mice models of disseminated mucormycosis. ABLC: amphotericin B lipid complex; L-AmB: liposomal amphotericin B; ANI: anidulafungin; CAS: caspofungin; CFU: colony forming unit; DKA: diabetic ketoacidosis; FMGX: fosmanogepix; ISA: isavuconazole; MICA: micafungin; POSA: posaconazole.

Combined Therapy	Mice Model of Mucormycosis and Method of Infection	Species of Mucorales	Efficacy on Survival Compared to Monotherapy	Efficacy to Decrease Organ CFU Compared to Monotherapy	Ref.
ABLC + CAS	-DKA - via the tail vein	*Rhizopus arrhizus* var. *delemar*	yes, improve survival	not better than ABLC alone (brain and kidney CFU)	[141]
L-AmB + ANI or MICA	-DKA -via the tail vein	*R. delemar* var. *delemar*	yes, improve survival	not better than L-AmB alone (kidney CFU)	[142]
L-AmB + MICA	-DKA -via the tail vein	*R. delemar* var. *delemar*	yes, improve survival	reduce kidney CFU	[142]
L-AmB + POSA	-DKA and neutropenic -via the tail vein	*R. arrhizus* var. *delemar*	no benefit	not better than L-AmB alone (brain and kidney CFU)	[144]
L-AmB + ISA	-neutropenic -intratracheally	*R. arrhizus* var. *delemar* and *M. circinelloides*	yes, improve survival	reduce lung and brain CFU	[143]
ISA + MICA	-neutropenic -intratracheally	*R. arrhizus* var. *delemar* and *M. circinelloides*	no benefit	not better then monotherapy (brain and kidney CFU)	[160]
L-AmB + FMGX	-neutropenic -intratracheally	*R. arrhizus* var. *delemar*	yes, improve survival	reduce lung and brain CFU	[145]
POSA + CotH3	-DKA -intratracheally	*R. arrhizus* var. *delemar*	yes, improve survival 100% survival	not better than monotherapy (lung and brain CFU)	[43]
L-AmB + CotH3	-DKA -intratracheally	*R. arrhizus* var. *delemar*	100% survival, trend to be better than L-AmB alone (*p* = 0.05)	not better than monotherapy (lung and brain CFU)	[43]

## Author Contributions

Conceptualization, F.D. and O.L. F.D. wrote the first draft of the manuscript. F.D., V.A. and D.G.-H. prepared the figures. All authors carefully reviewed the paper. All authors have read and agreed to the published version of the manuscript.

## Data Availability

Not applicable.
